# Supplementary material for: ATF3/SPI1/SLC31A1 Signaling Promotes Cuproptosis Induced by Advanced Glycosylation End Products in Diabetic Myocardial Injury
Source: Int J Mol Sci. 2023 Jan 14;24(2):1667. doi: 10.3390/ijms24021667 (PMC9862315; doi:10.3390/ijms24021667)
Supplement: Supplementary file 1 [file ijms-24-01667-s001.zip › ijms-2097701-supplementary.pdf]

Supplementary Material

1. **Supplementary Figure S1:**

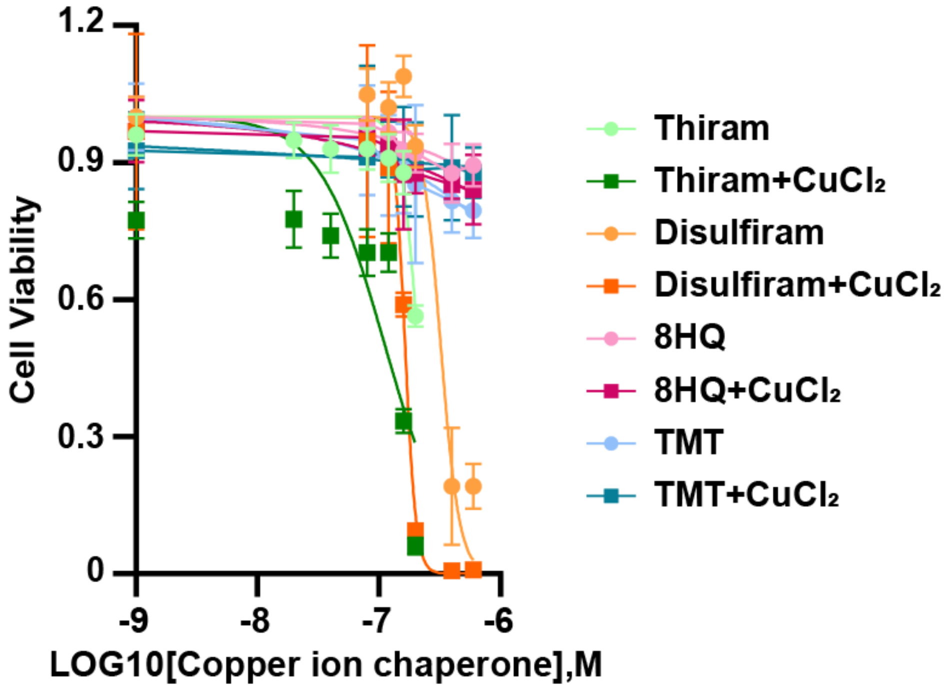

Supplementary Figure 1: Viability of AC16 cells after treatment with varied indicated copper ion chaperones with or without 10μM $CuCl_2$.

2. **Supplementary Figure S2**

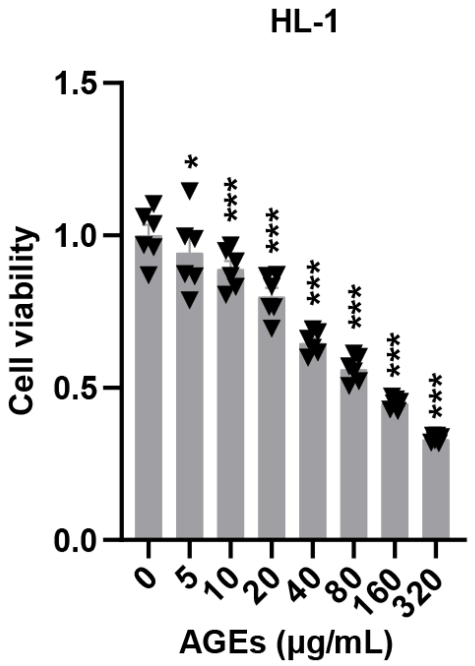

Supplementary Figure 2: Viability of HL-1 cells after treatment with AGEs of indicated concentrations.
